# Global output of research on epidermal parasitic skin diseases from 1967 to 2017

**DOI:** 10.1186/s40249-018-0456-x

**Published:** 2018-08-06

**Authors:** Waleed M. Sweileh

**Affiliations:** Department of Physiology, Pharmacology/Toxicology, Division of Biomedical Sciences, College of Medicine and Health Sciences, An-Najah National University, Nablus, Palestine

**Keywords:** Epidermal parasitic skin diseases, Bibliometric analysis, Keyword mapping, Scopus

## Abstract

**Background:**

Epidermal parasitic skin diseases (EPSD) occur in most countries and cause a considerable health and economic burden, particularly in the tropics and subtropics. The aim of this study was to assess and analyse peer-reviewed literature on EPSD in humans. The results of this study serve as an indicator of the extent the scientific community, health authorities, and international health agencies interact with EPSD as a health problem that is commonly associated with poverty and poor hygiene.

**Methods:**

A bibliometric analysis methodology was used. The Scopus database was used to retrieve documents about EPSD for the study period (1967–2017). The study focused on scabies, tungiasis, pediculosis, hookworm-related cutaneous larva migrans (HrCLM), myiasis, and cutaneous strongyloidiasis. Documents that specifically and explicitly discuss EPSD in animals, aquatic organisms, and birds were excluded.

**Results:**

In total, 4186 documents were retrieved. A fluctuated growth of publications on EPSD in the past five decades was found. The retrieved documents received 43 301 citations, an average of 10.3 citations per article and an *h*-index of 74. The keywords “scabies” and was the most commonly encountered keyword followed by the keywords “head lice” and “pediculosis”. The most active journal involved in publishing articles on EPSD was the *International Journal of Dermatology* (164; 3.9%). Researchers from 93 different countries published the retrieved articles. The USA led with 735 (17.6%) documents, followed by the UK (274; 6.5%), and Germany (259; 6.2%). In terms of institutions, the Charité – Universitätsmedizin Berlin in Germany was the most active in this field with 78 (1.9%) publications, followed by the Universidade Federal do Ceará in Brazil with 52 (1.2%) publications.

**Conclusions:**

Research on scabies and pediculosis dominated the field of EPSD research to the expense of tungiasis, HrCLM, myiasis, and cutaneous strongyloidiasis. There was an underrepresentation of literature from the tropics and subtropics despite EPSD being common in these areas. This could possibly be explained by the presence of limited number of non-English journals in the Scopus database. International research collaborations and research networking should be strengthened to help advance and prioritize research on EPSD.

**Electronic supplementary material:**

The online version of this article (10.1186/s40249-018-0456-x) contains supplementary material, which is available to authorized users.

## Multilingual abstracts

Please see Additional file 1 for translations of the abstract into five official working languages of the United Nations.

## Background

Epidermal parasitic skin diseases (EPSD) are a group of neglected parasitic diseases [1–5]. Important examples of EPSD include scabies, tungiasis, pediculosis, hookworm-related cutaneous larva migrans (HrCLM), myiasis, and cutaneous strongyloidiasis [6–11]. These EPSD have wide geographical distribution and are commonly associated with poverty and poor hygiene [1]. Therefore, EPSD are a public health issue, particularly for low- and middle-income countries [1, 2, 7, 12–16]. Future plans to control and minimize the health and economic burdens of EPSD require research activity in epidemiology, molecular biology, pharmacotherapy, and pathology. Therefore, the volume, evolution, visibility, and structure of scientific research on EPSD need to be investigated and made available for subsequent action by health policymakers.

The widely used and well-established methodology to measure the quantity and quality of research output on a certain scientific subject is bibliometric analysis, which is used to provide real and concrete data on research trends and priorities [17–19]. Bibliometric analysis is becoming an important, accessible, and widely accepted method to assess national and international research productivity, international collaboration, volume of citations, research trends, and scientific development in a particular field [20–24]. Bibliometric studies identify the direction of the research activity and thus enhance understanding of changes in any field [25]. Bibliometric analysis has been applied to malaria research [26], antibiotic resistance [27], cancer [22], diabetes [28], nutrition [26, 29, 30], and many other diseases and topics [31–33].

This study aimed to evaluate the volume of scientific publications related to EPSD in humans in order to shed more light on research related to this topic. In specific, the following objectives will be sought: (1) key countries, institutions, journals, and authors contributing to the topic, (2) annual number of publications, (3) most frequently encountered keywords, and (4) extent of international collaboration in EPDS research.

## Methods

### Bibliographic database

The study used a bibliometric analysis methodology. The study was limited to the period from 1967 to 2016. SciVerse Scopus, developed by Elsevier, was used to retrieve publications about the selected EPSD.

Scopus was selected for this study because it has several advantages over other databases such as Web of Science, Medline, and Google Scholar [34–37]. For example, Scopus is larger than Web of Science, more accurate than Google Scholar, 100% inclusive of MEDLINE. Therefore, all publications present in MEDLINE are already present in Scopus. The most important feature of Scopus is its ability to provide bibliometric indicators in a direct and simple way.

### Search strategy

The strategy of this study involved constructing a separate search strategy for each of the selected EPSD. Therefore, a search query was developed for the following components: (1) pediculosis (all types of lice infestation), (2) scabies, (3) tungiasis, (4) HrCLM, (5) myiasis, and (6) cutaneous strongyloidiasis. For each search query, a set of specific keywords were developed after reviewing the literature to obtain all possible keywords. Asterisks and quotation marks were used frequently to retrieve the maximum possible keywords. For example, the word tungiasis was entered as “cutaneous strongyloid*” where the asterisk was used as a wildcard while the quotation marks were used to limit the search to the exact phrase written. There were no language restrictions applied. A “title” search rather than a “title/abstract” search was implemented to increase accuracy and minimize false positive results. The title search would retrieve documents that are definitely in the field of EPDS. However, many documents might mention in the abstract any word related to EPDS as part of a list of infections present in a certain world region rather than focusing on EPDS. Therefore, the use of title search would lead to the minimum percentage of false positive. At the same time, the validation methodology adopted will ensure that the title search will also have minimum false negative results. In the final step, the results of the search queries were combined. Additional file 2 shows the strategy used and the keywords implemented in each search query.

### Exclusion and validation

An exclusion component was added to the overall search strategy to eliminate potential false positive results. The exclusion step included a collection of terms, phrases, and journal names that were found by manually checking the retrieved documents. All documents that specifically, directly, and explicitly discussed EPSD in animals, aquatic organisms, or birds were excluded. A validity check of the overall search strategy was done by comparing the number of retrieved documents for selected active authors with the number of publications obtained through a manual search for the same authors. The numbers of publications obtained by the two different methods were compared using interclass correlation. A *P-*value of less than 0.01 indicated significance and an interclass correlation of greater than 95% indicated high validity.

### Data analysis and visualization

In this study, the Hirsch index (*h*-index) was used to measure the impact of the publications. The *h*-index is defined as the number of articles (*n*) that have received at least *n* citations [38]. Graphs were created using the Statistical Package for Social Sciences (IBM SPSS statistics; version 21; Armonk, N.Y: IBM Corporation). VOSviewer software (version 1.6.8; Leiden University, the Netherlands) was used to create visualization maps [39–41].

## Results

### Types, languages, and subject areas of retrieved documents

In total, 4186 documents were retrieved. The majority were research articles (3055; 73.0%), followed by letters to the editor (443; 10.6%), review articles (379; 9.1%), notes (148; 3.5%), short surveys (87; 2.1%), conference papers (43; 1.0%), editorials (31; 0.7%). Twenty-eight different languages were encountered; the most common was English (3139; 75.0%), followed by French (221; 5.3%), German (211; 5.0%), and Spanish (195; 4.7%). A total of 3783 (90.4%) documents were published in journals indexed within the subject area of medicine, while 471 (11.3%) documents were published in journals within the subject area of immunology and microbiology, and 139 (3.3%) were published in journals within the subject area of agricultural and biological science. The total exceeds 100% due to potential overlap, as some journals could be indexed in more than one subject area. The retrieved documents included 219 (5.2%) epidemiological studies and 449 (10.7%) reported clinical case studies. The remaining were molecular or pharmacological, or clinical trials, or of unclassified type of study.

### Analysis of the growth of publications and citations

**Fig. 1:**
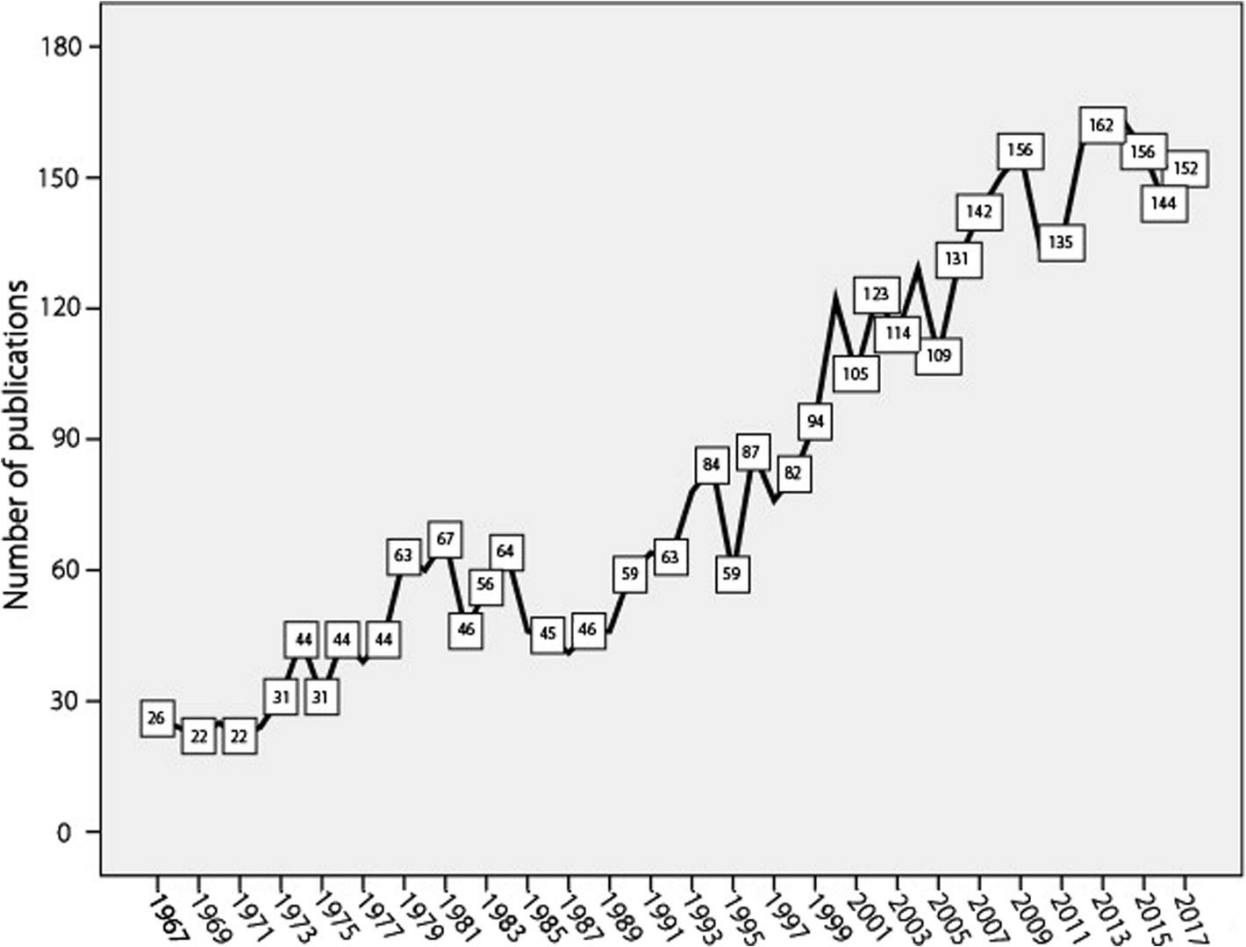
Growth of publications about EPSD (1967–2017)

The highest number of publications was recorded in 2014 with a total of 163 (3.9%) documents. Figure 1 shows the annual growth of publications during the past five decades. The total number of publications in the last decade (2008–2017) was 1509, which constitutes 36.0% of the retrieved documents.

The retrieved documents received 43 301 citations, an average of 10.3 citations per document and an *h*-index of 74. The highest number of citations recorded was 284 for a study published in the *New England Journal of Medicine* in 1995 [42]. The list of highly cited articles includes five research articles and five review articles (see Table 1). The research articles include one article in the field of molecular biology/genetics, three in the field of pharmacology and therapeutics, and one about scabies from a clinical practical point of view. All ten highly cited articles are about scabies and none were published in dermatology-related journals.

**Table 1:** Highly cited articles on EPSD (1967–2017)

Rank	Title	Reference	Year	Source title	Number of citations
1	The treatment of scabies with ivermectin	[42]	1995	*New England Journal of Medicine*	284
2	Scabies and pediculosis	[6]	2000	*The Lancet*	279
3	Genome sequences of the human body louse and its primary endosymbiont provide insights into the permanent parasitic lifestyle	[86]	2010	*Proceedings of the National Academy of Sciences of the United States of America*	243
4	Scabies	[87]	2006	*New England Journal of Medicine*	217
5	The body louse as a vector of reemerging human diseases	[88]	1999	*Clinical Infectious Diseases*	213
6	Scabies: a ubiquitous neglected skin disease	[9]	2006	*The Lancet Infectious Diseases*	182
7	Scabies	[4]	2006	*The Lancet*	169
8	Crusted scabies: Clinical and immunological findings in seventy-eight patients and a review of the literature	[89]	2005	*Journal of Infection*	159
9	First documentation of in vivo and in vitro ivermectin resistance in *Sarcoptes scabiei*	[90]	2004	*Clinical Infectious Diseases*	152
10	Permethrin and ivermectin for scabies	[91]	2010	*New England Journal of Medicine*	149

*EPSD* Epidermal parasitic skin diseases

### Most frequent author keywords

**Fig. 2:**
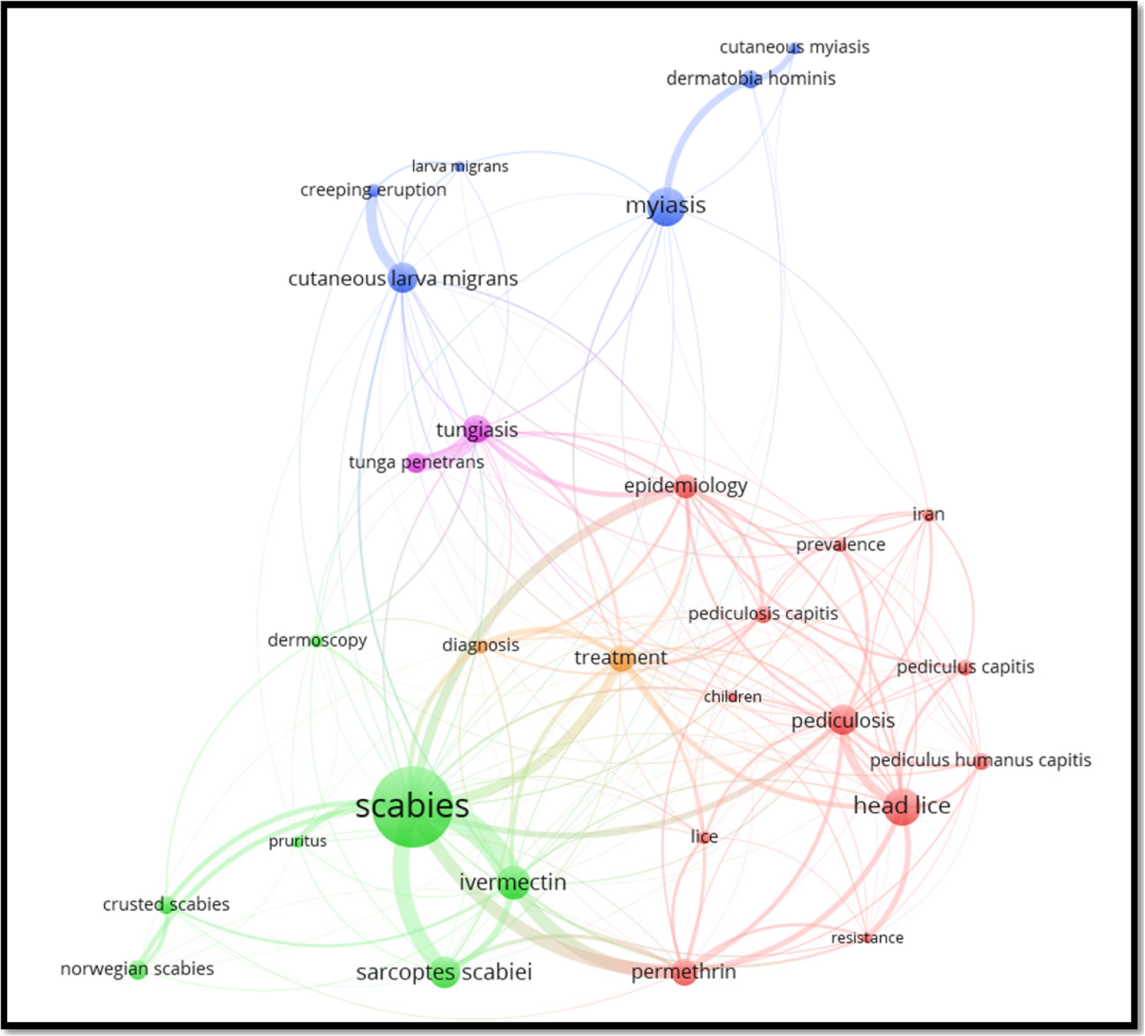
Mapping and clustering of author keywords related to EPSD (1967–2017). The first cluster (green) includes seven keywords, the second cluster (red) includes 12 keywords, the third cluster (blue) includes six keywords, the fourth cluster (purple) includes two keywords, and the fifth cluster (light orange) includes two keywords

Author keywords with minimum occurrences of 20 are visualized in Fig. 2. The plot includes 29 keywords distributed in five clusters. The keyword “scabies” has the largest node size indicating a high frequency relative to other keywords. The keyword lice was associated with the keyword treatment. And the keyword resistance.

### Most active journals

The most active journals involved in publishing articles on EPSD are summarized in Table 2. Eight of the ten most active journals are in the field of dermatology, while one is in the field of parasitology and one in general medicine. The total number of articles published by the ten most active journals was 755 (18.0%). The *International Journal of Dermatology* was the most productive (164; 3.9%), followed by *Archives of Dermatology* (122; 2.9%) and the *Journal of the American Academy of Dermatology* (94; 2.2%). However, publication in the *New England Journal of Medicine* received the highest number of mean citations per article (28.1), followed by those published in the *British Journal of Dermatology* (23.7).

**Table 2:** Most active journals publishing articles on EPSD (1967–2017)

Name of journal	Number of publications *N* = 4186	Percentage	Total citations received	Citations per article
*International Journal of Dermatology*	164	3.9	2520	15.4
*Archives of Dermatology*	122	2.9	2357	19.3
*Journal of the American Academy of Dermatology*	94	2.2	2132	22.7
*British Journal of Dermatology*	66	1.6	1566	23.7
*Annales de Dermatologie et de Vénéréologie*	65	1.6	302	4.6
*Parasitology Research*	62	1.5	1119	18.0
*Pediatric Dermatology*	54	1.3	1000	18.5
*Korean Journal of Dermatology*	46	1.1	98	2.1
*Cutis*	42	1.0	450	10.7
*New England Journal of Medicine*	40	1.0	1122	28.1
Total	755	18.0		

*EPSD* Epidermal parasitic skin diseases

### Most active countries

Researchers from 93 different countries published the retrieved articles. Table 3 shows a list of the ten most active countries. The USA led with a total of 735 (17.6%) documents, followed by the UK (274; 6.5%) and Germany (259; 6.2%). The total research output of these countries was 2445, which constitutes 58.4% of the worldwide research output. The ten most active countries include five countries in Western Europe, two in the Western Pacific Region, one in North America, one in South America, and one in Southeast Asia.

**Table 3:** Most active countries publishing articles on EPSD (1967–2017)

Rank	Country	Number of publications *N* = 4186	Percentage
1	USA	735	17.6
2	UK	274	6.5
3	Germany	259	6.2
4	France	250	6.0
5	Italy	211	5.0
6	Australia	190	4.5
7	Brazil	175	4.2
8	Spain	129	3.1
9	India	120	2.9
10	Japan	102	2.4
	Total^a^	2445	58.4%

*EPSD* Epidermal parasitic skin diseases

^a^There might be some overlap in the numbers due to international research collaboration. Therefore, the exact total number of publications might be less than what is calculated

### Most active institutions and authors

Researchers from different academic institutions participated in publishing the retrieved articles. The highly active institutions/organizations are shown in Table 4. Charité – Universitätsmedizin Berlin in Germany was the most productive in this field with 78 (1.9%) publications, followed by the Universidade Federal do Ceará in Brazil with 52 (1.2%) publications. The list includes five institutions in Australia, two in Germany, two in Italy, two in France, and one in Brazil.

**Table 4:** Most active institutions publishing EPSD research (1967–2017)

Rank^a^	Institution/Organization	Frequency *N* = 4186	Percentage	Country
1	Charité – Universitätsmedizin Berlin	78	1.9	Germany
2	Universidade Federal do Ceará	52	1.2	Brazil
3	Menzies – School of Health Research	47	1.1	Australia
4	Queensland Institute of Medical Research	42	1.0	Australia
4	Università degli Studi di Milano	42	1.0	Italy
4	University of Queensland	42	1.0	Australia
7	James Cook University	41	1.0	Australia
8	IRCCS^b^ Foundation Rome	34	0.8	Italy
9	Charles Darwin University	30	0.7	Australia
10	Inserm	25	0.6	France
10	Heinrich-Heine-University Dusseldorf	25	0.6	Germany
10	Aix-Marseille Université	25	0.6	France
	Total^c^	483	11.5	

*EPSD* Epidermal parasitic skin diseases

^a^Equal countries have the same ranking number, and then a gap is left in the ranking numbers

^b^“Istituto di Ricovero e Cura a Carattere Scientifico”

^c^There might be some overlap in the numbers due to international research collaboration. Therefore, the exact total number of publications might be less than what is calculated

**Fig. 3:**
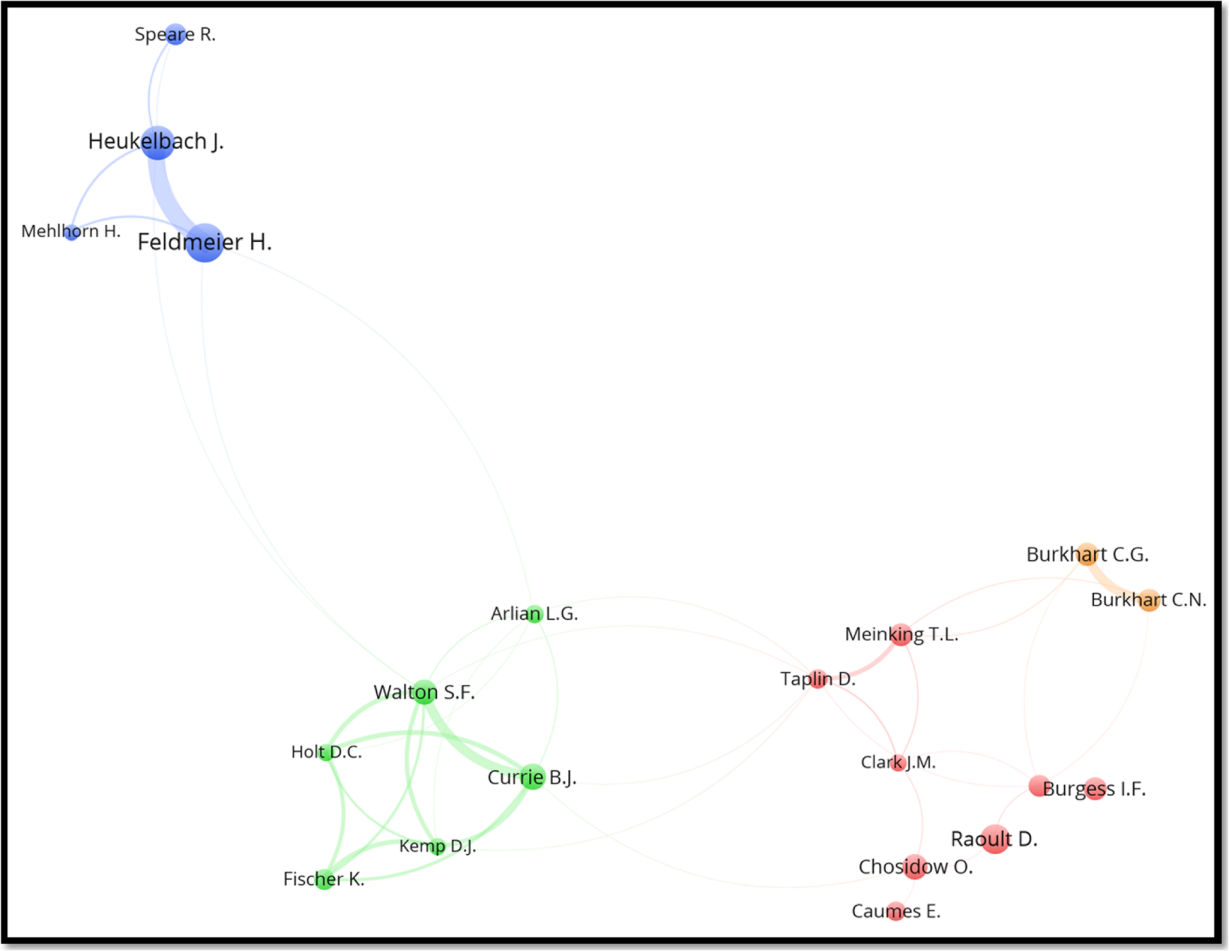
Mapping and clustering of active authors writing on EPSD (1967–2017). Both Veraldi, S and Parish, L.C are not shown in the map as they are not part of any research network present in the map. The map shows 20 authors distributed in six clusters with each cluster representing a network of collaborating researchers. The sizes of the nodes in the map are proportional to the number of publications

A total of 11 122 authors participated in publishing the retrieved articles, giving an average of 2.7 authors per article. Authors with a minimum productivity of 15 documents are visualized in Fig. 3. The figure shows the names of the most active authors and their research networking and collaboration. Each cluster of researchers is considered a network of collaborating authors while the thickness of connecting lines and distance between authors represent extent of research collaboration.

## Discussion

This study analysed literature on EPSD published in the past five decades. The results showed a slow growth of publications in this field and limited contribution of several important world regions characterized by poverty and low health standards. Epidemiological studies in this field are also limited in number, which adds to the neglect of the research community and health policymakers toward these diseases. The fact that the retrieved literature related to EPSD had an *h*-index of 74 indicates that this topic receives an inadequate attention and the number of interested people in this topic is limited relative to other topics [43, 44]. Several published studies have pointed out that these diseases are neglected at the global scientific level [1, 45–48]. Calls for more studies and research on this topic are needed, particularly from low- and middle-income countries. Such calls have been made by other researchers several years ago [1]. For example, Feldmeier H and Heukelbach J in 2008 recommended further epidemiological research to get reliable data on spatial distribution, incidence, prevalence, seasonal variation, and clustering of different EPSD in the same population [1].

The ten highly cited articles found in this study are about scabies and none are about pediculosis, tungiasis, myiasis, or HrCLM. No explanation could be provided for this higher citation for publications in scabies relative to other EPDS. However, the nature of the disease which affects all the body and potential effective therapies might have played a role in the difference in number of citations for documents in scabies versus pediculosis. It is estimated that about 1–10% of the world’s population is infected with scabies, and in certain crowded areas with unhygienic conditions the prevalence might be as high as 50% [49, 50]. Pediculosis is also a common infestation, particularly among children. The figure showing author keywords indicates that the number of publications in pediculosis/head lice is close to that of scabies as shown by the node size. Pediculosis has been reported from both low- and high-income countries such as the USA, UK, France, Australia, Cameron, Brazil, Benin, Iran, Pakistan and Denmark [51–54]. Tungiasis is present in tropical and subtropical regions of the world, particularly in South America, where poor communities bear the highest burden, with a prevalence rate as high as 80% in certain communities [13]. It is expected that more than 20 million individuals are at risk in the Americas alone. Myiasis is an infection of a fly larva (maggot) in human tissue [55]. It is similar to tungiasis in that it is common in the tropics and subtropics of Africa and the Americas [55]. In terms of HrCLM, the incidence is more common in developed countries where it is considered second to pinworm among helminth infections [56–59]. It is common in warm climates where people tend to walk barefoot and come into contact with animal feces [57–59]. The high incidence of tungiasis, myiasis, and HrCLM explains why Brazil is one of the top active countries (and with a top active institution) for research output. Travelers from tropical countries such as ones in South America to the USA might be one possible reason for the incidence of EPSD in the USA [60–62]. Cutaneous strongyloidiasis was not found to be a frequent author keyword. This condition seems to be relatively underresearched and rare. Strongyloidiasis presents mainly as gastrointestinal symptoms and rarely as a cutaneous manifestation. Such clinical manifestations are seen in immunocompromised patients [63–68].

The study showed that the USA published the largest volume of literature EPSD. However, none of the ten most active institutions are based in the USA. German and Australian institutions dominated this field. Actually, the most prolific author in this field is affiliated with a German institution, while the second most prolific author is based in an Australian institution, with strong collaborations between researchers in these two institutions. Brazil ranked among the ten most active countries and the Brazilian institution Universidade Federal do Ceará also ranked among the ten most active institutions. Published studies indicated that HrCLM and tungiasis are endemic and result in high mortality and morbidity in Brazil [56, 69–71].

One of the most frequently encountered author keywords in this study was “treatment” as associated with the keyword “lice”. Resistance to insecticides used for the treatment of lice infestation has been reported in the last decade [72–81]. Studies have indicated that the prevalence of insecticide resistance in head lice is caused by knockdown resistance-type mutations [74, 77, 81]. Failure of treatment due to resistance might create a problem in finding an effective and convenient treatment method, as combing might not be suitable in all households. The results also show that one of the most highly cited articles on ESPD is about the emergence of resistance in scabies mites to ivermectin. The emergence of resistance to common therapies in addition to the neglected nature of these diseases give a warning to researchers, policymakers, and healthcare providers to direct their efforts toward eradicating these diseases, especially in countries with limited resources.

The study found that research collaboration and networking on EPSD research was limited. This can be concluded from the fact that the 15 active researchers on this topic were distributed in six different research clusters with thin, weak, connecting lines between clusters. Neglected health conditions require more research networking and collaboration among researchers in different institutions and different countries. Such networking and collaboration might serve as an international platform for scientists and healthcare providers to share opinions and ideas regarding prevention and eradication of these diseases.

This study had a few limitations that are similar to those mentioned in bibliometric studies [44, 82–85]. One important limitation is the fact that many health-related journals in developing countries might not be indexed in Scopus, as is the case of Iranian, Korean, and African journals, and therefore some literature might have been missed. It should be emphasized that more than 95% of the journals indexed in Scopus are in English, with the database favoring journals published in North American and European countries. At the same time, the vast majority of journals published in Asia in a non-English language are not indexed in Scopus. This is true drawback in dealing with Scopus or any other database created in the USA or Europe, and suggests that data presented in the current study might underestimate the research output from countries other than those in North America and Europe. Another potential limitation are the keywords used, which night not be 100% comprehensive and therefore some of the publications might have been missed. A potentially important limitation is the research collaboration among certain researchers, which created a bias in top active authors toward those who exist within research team and networks. For example, when two researchers collaborate and publish together then the research output of the two authors will be counted separately based on bibliometric methodology despite the presence of overlap in their research output. Researchers who do not exist within research networks might not appear in maps showing the most active authors. Therefore, data in the current study pertaining to active researchers and institutions need to be carefully interpreted and must take into consideration the drawbacks of Scopus and the concept of self-citation, as well as the existence of authors in active research groups which might affect the ranking and names shown in the list of active authors and institutions. For example, self-citations is known to increase the *h*-index and being within an active research group will increase the research output of all the authors in the group regardless of the size of their role in the publication. Finally, it should be emphasized that in this study, articles that explicitly discussed EPSD in non-humans were excluded. If such articles were not excluded, the results will be different to the ones presented here.

## Conclusions

This is the first-ever bibliometric analysis of peer-reviewed literature related to EPSD. The results presented in this study are useful for people interested in advancing research in this field. The study showed that research on EPSD does not superimpose with the epidemiology patterns of EPSD, particularly in tropical regions of Africa, South America, and Southeast Asia. International research collaborations and research networks should be strengthened in this field to help prioritize and advance research on EPSD. Finally, the results are also useful for funding agencies, donors, and other international health agencies interested in promoting healthcare among poor communities.

## Additional files

Additional file 1: Multilingual abstracts in the five official working languages of the United Nations. (PDF 243 kb)

Additional file 2: Search strategy and keywords. The file includes keywords used in the search query as well as the keywords used in the exclusion step. (DOCX 13 kb)

## Abbreviations

EPSD: Epidermal parasitic skin diseases
HrCLM: Hookworm-related cutaneous larva migrans
