# Supplementary material for: Global output of research on epidermal parasitic skin diseases from 1967 to 2017
Source: Infect Dis Poverty. 2018 Aug 6;7:74. doi: 10.1186/s40249-018-0456-x (PMC6091169; doi:10.1186/s40249-018-0456-x)
Supplement: Supplementary file 1 — Multilingual abstracts in the five official working languages of the United Nations. (PDF 243 kb) [file 40249_2018_456_MOESM1_ESM.pdf]

Translation of the abstract into the five official working languages of the United Nations

الناتج العالمي للبحوث المتعلقة بالأمراض الجلدية الطفيلية في البشرمن عام 1967 حتى عام 2017

وليد م. صويلح

الملخص

الخلفية: تحدث الأمراض الجلدية البشرية الطفيلية (EPSD) في معظم البلدان وتسبب عبئا صحيا واقتصاديا كبيرا ، خاصة في المناطق الاستوائية وشبه الاستوائية. الهدف من هذه الدراسة هو تقييم وتحليل الدراسات التي خضعت لاستعراض الأقران حول الأمراض الجلدية الطفيلية في البشر. تعتبر نتائج هذه الدراسة بمثابة مؤشر على مدى تفاعل المجتمع العلمي والسلطات الصحية ، والوكالات الصحية الدولية مع الأمراض الجلدية البشرية الطفيلية باعتبارها مشكلة صحية ترتبط عادة بالفقر وقلة النظافة.

الطرق: تم استخدام منهجية التحليل البيبليومتري. تم استخدام قاعدة بيانات سكوبس لاسترداد الوثائق حول الأمراض الجلدية البشرية الطفيلية لفترة الدراسة من 1967 الى 2017. وركزت الدراسة على الجرب ، والتنجيات ، والقمل ، وداء اليرقات الجلدي المرتبط بالدودة الشصية (HrCLM) ، وداء الدودة الحلزونية، وداء الأسطوانيات الشعرية الجلدية. تم استبعاد الوثائق التي تناقش بشكل واضح وصريح الأمراض الجلدية الطفيلية في الحيوانات ، والكائنات المائية ، والطيور.

النتائج: في المجموع ، تم استرجاع 4186 وثيقة. وجد بأن هناك نموا متذبذبا في المنشورات حول الأمراض الجلدية الطفيلية في العقود الخمسة الماضية. تلقت الوثائق المستردة 43301 استشهاداً ، بمتوسط 10.3 استشهادات لكل مقالة ومؤشر *h* ل 74. كانت الكلمة المفتاحية "الجرب" هي الأكثر شيوعا تبعتها كلمات "قمل الرأس" و "التقمل". كانت المجلة الأكثر نشاطًا في نشر المقالات حول الأمراض الجلدية الطفيلية هي *المجلة الدولية للأمراض الجلدية* (164 ؛ 3.9٪). قام باحثون من 93 دولة مختلفة بنشر المقالات التي تم استرجاعها. وأظهر التوزيع الجغرافي أن دول الشرق الأوسط وأفريقيا وأوروبا الشرقية لديها أقل مخرجات البحوث ، في حين أن أمريكا الشمالية وأوروبا الغربية كان لديها أعلى مخرجات البحوث. كان في الصدارة الولايات المتحدة بما يمثل 735 (17.6٪) ، تليها المملكة المتحدة (274 ؛ 6.5٪) ، وألمانيا (259 ؛ 6.2٪). من حيث المؤسسات ، كانت *جامعة - شاريتيه للطب الجامعي ببرلين* في ألمانيا الأكثر نشاطًا في هذا المجال من خلال 78 منشورا (1.9٪) ، تليها *جامعة سيارا الاتحادية في البرازيل* مع 52 منشورا (1.2٪).

الاستنتاجات: سادت البحوث على الجرب والقمل في مجال البحث حول الأمراض الجلدية الطفيلية على حساب التنجيات ، وداء اليرقات الجلدي المرتبط بالدودة الشصية (HrCLM) ، وداء الدودة الحلزونية، وداء الأسطوانيات الشعرية الجلدية. كان هناك نقص في تمثيل المنشورات من المناطق الاستوائية وشبه الاستوائية على الرغم من أن الأمراض الجلدية الطفيلية شائعة في هذه المناطق. يمكن تفسير ذلك من خلال وجود عدد محدود من المجلات غير الإنجليزية في قاعدة بيانات سكوبس. يجب تعزيز التعاون البحثي الدولي وإقامة الشبكات البحثية للمساعدة في تطوير البحوث على مستوى الأمراض الجلدية الطفيلية وتحديد أولوياتها.

Translated from English version into Arabic by Rand Gharaibeh and Free bird, through

TRANSLATORS WITHOUT BORDERS

1967–2017 年全球表皮寄生虫皮肤病研究成果

Waleed M. Sweileh

摘要

引言：表皮寄生虫皮肤病(EPSD)在大多数国家均有病例，特别是在热带和亚热带地区，并可造成严重的健康和经济负担。本研究旨在评估和分析经同行评议的人类 EPSD 相关文献。这

项研究的结果可以作为科学家、卫生管理者和国际卫生机构处理 EPSD 健康问题的参照科学指标，进而改善贫穷和不良卫生状况。
方法：本研究采用文献计量分析方法，从 Scopus 数据库检索 1967–2017 年的 EPSD 相关文献。本研究主要纳入的病种为疥疮、潜蚤病、虱病、钩虫相关的皮肤幼虫(HrCLM)、蝇蛆病和皮肤线虫病，并将明确讨论动物、水生生物和鸟类 EPSD 的文献排除在外。
结果：本研究总共检索出 4186 篇文献。在过去的五十年中， EPSD 相关出版物呈波动增长趋势。所检索到的文献总被引数为 43 301 次，平均被引频次为 10.3，h 指数为 74。最常见的关键词是“疥疮”，其次是“头虱”和“头虱症”。《国际皮肤病学杂志》是发表有关 EPSD 的文章最活跃的期刊(164; 3.9%)。上述文章的作者来自 93 个国家。地理分布显示，中东、非洲和东欧国家的研究产出最少，而北美和西欧的研究产出最高。美国以发表 735 篇(17.6%)文章居首，其次是英国(274 份;6.5%)和德国(259;6.2%)。在机构方面，德国柏林的 Charite - Universitatsmedizin 在这一领域最为活跃，发表文章数为 78 篇(1.9%)；紧随其后的是巴西 Ceara 大学，为 52 篇(1.2%)。
结论：对比研究费用可得知，相对于潜蚤病、HrCLM、蝇蛆病和皮肤圆线虫病，疥疮和虱病是 EPSD 研究的重点。尽管 EPSD 在热带和亚热带很常见，但是来自这些地区的研究文献却缺乏代表性，这可能是由于在 Scopus 数据库收录的非英语期刊较少。因此，应加强国际研究合作和研究网络建设，帮助促进和优先开展 EPSD 研究。

Translated from English version into Chinese by Xin-Yu Feng, edited by Jin Chen

Production mondiale de recherches sur les parasitoses épidermiques de 1967 à 2017.

Waleed M. Sweileh

Résumé
Contexte : Les parasitoses épidermiques sont présentes dans la plupart des pays et constituent un fardeau économique et sanitaire considérable, en particulier dans les régions tropicales et subtropicales. L'objectif de cette étude était d'évaluer et d'analyser la littérature sur les parasitoses épidermiques humaines publiée dans les revues à comité de lecture. Ses résultats indiquent dans quelle mesure la communauté scientifique au sens large, les autorités sanitaires et les agences sanitaires internationales s'intéressent au problème sanitaire des parasitoses épidermiques, fréquemment associé à la pauvreté et à une mauvaise hygiène.
Méthodes : Une méthodologie d'analyse bibliométrique a été utilisée. La base de données Scopus a été utilisée pour collecter les documents traitant de parasitoses épidermiques sur la période étudiée (1967-2017). L'étude se concentre sur la gale, la tungose, la pédiculose, la larva migrans cutanée (LMC), les myiases et la larva currens (infestation cutanée par des strongyloïdes). Les documents traitant spécifiquement et explicitement de parasitoses épidermiques chez les animaux, les organismes aquatiques et les oiseaux ont été exclus.
Résultats : Au total, 4186 documents ont été retenus. Une croissance fluctuante du nombre de publications consacrées aux parasitoses épidermiques a été constatée au cours des cinquante dernières années. Les documents retenus ont été cités 43 301 fois, avec une moyenne de 10,3

citations par article et un indice *h* de 74. Le mot-clé « gale » est le plus fréquemment rencontré, suivi de « poux » et « pédiculose ». La revue la plus active pour la publication d'articles sur les parasitoses épidermiques était l'*International Journal of Dermatology* (164 ; 3,9 %). Les articles retenus ont été publiés par des chercheurs de 93 pays différents. La répartition géographique a montré que les pays du Moyen-Orient, d'Afrique et d'Europe de l'Est avaient la plus faible production de recherches et l'Amérique du Nord et l'Europe de l'Ouest la production la plus élevée. Les États-Unis sont en tête avec 735 documents (17,6 %), suivis par le Royaume-Uni (274 ; 6,5 %) et l'Allemagne (259 ; 6,2 %). En ce qui concerne les institutions, l'hôpital universitaire de la Charité, à Berlin en Allemagne, était le plus actif dans ce domaine avec 78 publications (1,9 %), suivi par l'Université fédérale du Ceará au Brésil, avec 52 publications (1,2 %).

Conclusions : Les travaux sur la gale et la pédiculose dominent les recherches sur les parasitoses épidermiques au détriment de la tungose, de la LMC, de la myiase et de la larva currens cutanée. Les auteurs des régions tropicales et subtropicales étaient sous-représentés, malgré la fréquence des parasitoses épidermiques dans ces parties du monde. Cela pourrait s'expliquer par la présence limitée des revues non anglophones dans la base de données Scopus. Il y aurait lieu de renforcer les collaborations de recherche internationales et les réseaux de recherche afin d'aider à faire progresser les recherches sur les parasitoses épidermiques et à en faire une priorité.

Translated from English version into French by Soline Le Saux and Suzanne Assenat, through

TRANSLATORS
WITHOUT BORDERS

Завезённые паразитарные болезни в континентальном Китае: текущее положение и перспективы по улучшению контроля и профилактики

Лань-Гуй Сун, Син-Да Цзэн, Янь-Ся Ли, Бэй-Бэй Чжан, Сяо-Ин У, Дун-Цзюань Юань, Ай Хэ и Чжун-Дао У

Аннотация

Справочная информация: Высокая заболеваемость паразитарными болезнями в развивающихся странах ежегодно приводит к миллионам смертей и инвалидностей. Китай также существенно пострадал от паразитарных инфекций, в том числе от филяриатоза, лейшманиоза, малярии, шистосомоза, а также передаваемых через почву нематодоз. Однако в результате укрепления усилий по всеобъемлющему контролю за паразитарными болезнями ситуация в Китае значительно улучшилась, что привело к искоренению филяриатоза в 2006 году и к существенному контролю за другими заболеваниями. Тем не менее, случаи завезённых паразитарных болезней являются неизбежными, а в результате растущей глобализации и регионального сотрудничества всё чаще поступает информация о таких случаях. Данные завезённые болезни представляют собой основное препятствие на пути устранения нескольких паразитарных заболеваний, таких как малярия.

Основная часть: В настоящей работе рассматриваются завезённые случаи паразитарных болезней в континентальном Китае, в особенности малярии и шистосомоза, на основании данных, отдельно представленных в ежегодных обзорах по Китаю, а также в других научных публикациях. Мы подвели итоги по новым сложностям, с которыми сталкиваются усилия по контролю за паразитарными болезнями в континентальном Китае, а также по перспективам относительно улучшения контроля. Мы считаем, что необходимы как обеспечение профессионального обучения, так и обновлённая подготовка медицинского персонала, а также управление и надзор за лицами, въезжающими в Китай. Более того, мы предлагаем считать китайских трудовых мигрантов приоритетной группой для целей медико-санитарного просвещения, а также подчёркивать необходимость информирования общественности относительно завезённых болезней. Кроме того, мы особо отмечаем важность изучения распространения представленных/потенциальных переносчиков заболеваний, восприимчивости к паразитам, а также улучшения методов диагностики и запасов лекарственных препаратов.

Выводы: Завезённые случаи представляют собой основную сложность на пути к искоренению нескольких паразитарных заболеваний, таких как малярия и шистосомоз, в континентальном Китае. Китай должен направить свои действия на устранение указанных сложностей, которые тесно связаны с общенациональной биологической безопасностью.

Translated from English version into Russian by Liudmila Tomanek and Anna Romanenko, through

TRANSLATORS
WITHOUT BORDERS

Resultado global de la investigación sobre las parasitosis epidérmicas desde 1967 hasta 2017

Waleed M. Sweileh

Resumen

Contexto: Las parasitosis epidérmicas (PE) ocurren en muchos países y causan importantes cargas económicas y sanitarias, especialmente en los trópicos y subtrópicos. El objetivo de este estudio ha sido evaluar y analizar los escritos de revisión por pares sobre las PE en seres humanos. Los resultados de este estudio sirven como indicador del alcance de la interacción de la comunidad científica, las autoridades sanitarias y las agencias internacionales para la salud con las PE como un problema de salud comúnmente asociado a la pobreza y la mala higiene.

Metodología: Se utilizó una metodología de análisis bibliométrica. La base de datos Scopus se usó como fuente de documentos sobre las PE para el periodo de estudio (1967-2017). El estudio se centró en la sarna, tungiasis, pediculosis, larva migrans cutánea por anquilostoma (LMCpA), miasis y estrongiloidasis cutánea. Se excluyeron los documentos que específica o explícitamente trataban sobre las PE en animales, organismos acuáticos y aves.

Resultados: En total, se incluyeron 4186 documentos. Se apreció un aumento fluctuante de las publicaciones sobre las PE en las últimas cinco décadas. Los documentos incluidos contenían 43

301 citas, una media de 10,3 citas por artículo y un índice *h*de 74. La palabra clave "sarna" fue la palabra clave más utilizada seguida de "piojos" y pediculosis". La revista más activa en publicar artículos sobre las PE fue la *International Journal of Dermatology* (164; 3,9%). Investigadores de 93 países diferentes publicaron los artículos seleccionados. Una distribución geográfica mostró que los países de Oriente Medio, África y Europa del Este obtuvieron los resultados de investigación más bajos, mientras que en América del Norte y Europa Occidental los resultados fueron los más altos. Los EEUU lideraron con 735 (17,6%) documentos, seguido de Reino Unido (274; 6,5%) y Alemania (259; 6,2%). En términos de instituciones, el Charité - Universitätsmedizin Berlin en Alemania fue la más activa en este campo con 78 (1,9%) publicaciones, seguido por la Universidade Federal do Ceará en Brasilcon 52 (1,2%) publicaciones.

Conclusiones: La investigación sobre la sarna y la pediculosis dominó en el campo de investigación de las PE en detrimento de la tungiasis, LMCpA, miasis y la estrongiloidasis cutánea. Hubo una escasa representación escrita de los trópicos y subtrópicos, a pesar de que las PE se encuentran con frecuencia en estas áreas. Esto se podría explicar por la presencia de un número limitado de revistas no anglófonas en la base de datos Scopus. Las colaboraciones internacionales y las redes de investigación deberían fortalecerse para ayudar el desarrollo y priorizar la investigación de las PE.

Translated from English version into Spanish by María Galonce Terán and Maria Alejandra Aguada, through

TRANSLATORS
WITHOUT BORDERS
