# Supplementary material for: Global output of research on epidermal parasitic skin diseases from 1967 to 2017
Source: Infect Dis Poverty. 2018 Aug 6;7:74. doi: 10.1186/s40249-018-0456-x (PMC6091169; doi:10.1186/s40249-018-0456-x)
Supplement: Supplementary file 2 — Search strategy and keywords. The file includes keywords used in the search query as well as the keywords used in the exclusion step. (DOCX 13 kb) [file 40249_2018_456_MOESM2_ESM.docx]

**Additional file 1**

**Search strategy and keywords**

| Item number | Function | Topic | keywords |
| --- | --- | --- | --- |
| 1 |  | Scabies | TITLE(Scabie* OR scabic* OR "Sarcoptes Scabiei") AND NOT TITLE(Streptomyces)) |
| 2 | OR | Pediculosis | (TITLE(pediculosis OR "head lice" OR "body lice" OR "pubic lice" OR "lice infestation*" OR "louse infestation*" OR pediculus) OR (TITLE(louse) AND TITLE-ABS(infestation* OR infection))) |
| 3 | OR | HrCLM | TITLE("Cutaneous larva migrans")) |
| 4 | OR | Tungiasis | (TITLE(Tungia*) OR (TITLE("sand flea") AND TITLE-ABS(Tungia*))) |
| 5 | OR | Myasis | (TITLE(Myiasis) |
| 6 | OR | General terms | TITLE( ("parasitic skin infestation*") OR ("Epidermal parasitic skin") OR ("parasitic skin disease*") OR ("parasit* skin disease") OR ("parasitic skin infect*")) |
| 7 | OR | Cutaneous strongyloidiasis | ( ( ( TITLE ( strongyloid* )  AND  TITLE ( skin  OR  cutaneous  OR  epiderm* )  AND NOT  TITLE ( penetrating )  AND  TITLE-ABS ( infect* ) )  AND  PUBYEAR  <  2018  AND NOT  TITLE-ABS ( dog  OR  cat  OR  animal ) ) ) |
|  |  | **Combine results of 1, 2, 3, 4, 5, 6 and 7** | |
| 8 | Exclude | AND NOT TITLE (avian OR sea* OR ocean OR river OR Salmon OR Turkey* OR seal OR *nasal* OR vaginal OR alveolar OR fly OR marine OR salamon OR *ophthal* OR dog* OR poultry OR rabbits OR intestinal OR donkey OR deer OR oral OR *ocular OR cats OR buffalo OR pigs OR cattle OR sheep OR dog OR lizard OR bird OR fish OR salamon OR animal OR ecolog* OR sea OR marine OR sheep OR veter* OR urinary OR crustacea OR entomolog* OR eco* OR forest OR wildlife OR "fresh water" OR plant)) AND NOT (SRCTITLE(Endo* OR sea OR ocean OR river OR waterbirds OR evolution OR wildlife OR Poultry OR Acar* OR insect* OR Veter* OR Opthal* OR entomo* OR water OR bird OR fish OR Aqua* OR wlidlife OR zoo* OR inverteb* OR agricul* OR animal*)))) | |
| 9 | Limit period | 1967 - 2017 | |
| 10 | Limit source type | Journal articles only | |
|  |  |  | |
